# GMP compliant simplified fast and high yielding automated synthesis of [^18^F]fallypride without the need of HPLC purification

**DOI:** 10.1186/s41181-025-00343-w

**Published:** 2025-05-04

**Authors:** Ammar Alfteimi, Yi Zhao, Ulf Lützen, Alexander Helm, Michael Jüptner, Maaz Zuhayra

**Affiliations:** Department of Nuclear Medicine, Molecular Diagnostic Imaging and Therapy, University Hospital of Schleswig-Holstein (UKSH), Campus Kiel, Karl Lennert Cancer Center North, Feld-Str. 21, D-24105 Kiel, Germany

**Keywords:** Automated radiosynthesis, Fallypride, D2/D3 receptors, F-18

## Abstract

**Background:**

[^18^F]Fallypride PET has been used to study D2/3 receptor occupancy and density in neuropsychiatric disorders including Huntington’s disease (HD) and aging in humans. Nevertheless, the various synthetic methods including those provided by commercial synthesizers for [^18^F]fallypride exhibit a disadvantage concerning the necessity of using a HPLC purification step, which causes difficulties in the automation, leads to long synthesis times and moderate yields. Therefore utilizing the purification step by SPE cartridges is considered highly desirable for future commercialization of radiopharmaceutical cassettes. In our lab we have developed a simplified reliable automatic Radiosynthesis of [^18^F]fallypride by using SPE cartridges for the purification step without the need of HPLC.

**Results:**

A simplified Radiosynthesis of [^18^F]fallypride has been developed without the use of HPLC for both a commercial cassette based synthesis system (AllinOne (AiO) system, Trasis, Belgium) and a research synthesis module with fixed tubing (RNplus, Synthra, Germany). The cleaning step involves a serial combination of several SPE cartridges. The synthesis time was shortened by 44% compared to synthesis using HPLC. At the same time the not decay corrected yield increases from 44 to 59% by using TBAHCO_3_ as phase transfer catalysts and from 17 to 31% for the synthesis with K_2_CO_3_/Kryptofix-[2.2.2] compared to synthesis using HPLC. The Radiochemical purity was always > 98% and all quality control parameters (e.g. sterility, endotoxin, stability and Radiochemical purity) conformed with requirements of the European Pharmacopoeia.

**Conclusions:**

A GMP compliant automatic synthesis of [^18^F]fallypride including purification using simple solid phase extraction cartridges instead of HPLC was developed and evaluated. The implementation of the simplified synthesis in both used commercial modules allows efficient and reproducible Radiosynthesis of [^18^F]fallypride and leads to short synthesis times and high radiochemical yields with high radiochemical purity.

## Background

[^18^F]fallypride is a widely used positron emission tomographic (PET) radio tracer for imaging the dopaminergic system in the brain (Honer et al. 2004; Slifstein et al. 2004). It is a selective, high-affinity antagonist for D2/D3 receptors and has been employed to measure striatal and extrastriatal D2/D3 receptor. Its high specific in vivo uptake in brain regions containing DA D2/3 receptors as well as the reversibility of its in vivo binding to the receptors allows quantitation of their levels in striatal and extrastriatal brain regions using reversible kinetic modeling methods (Christian et al. 2009; Constantinescu et al. 2011; Mukherjee et al. 1999; Siessmeier et al. 2005).

The first approach for the synthesis of [^18^F]fallypride was reported by Mukherjee and coworkers, performing manual synthesis(Mukherjee et al. 1995) The radiochemical yields were between 20% and 40% (decay corrected) with 33–63 GBq/mmol of molar activity and relatively long reaction time of about 30 min. Moreover, a cleaning step had to be introduced, which takes up to additional 30 min. Later, a fully automated synthesis of [^18^F]fallypride by using the synthesizer TracerLab FX-FN (GE Healthcare) was proposed by Ansari et al. (Ansari et al. 2006) They could show that [^18^F]fallypride can be successfully carried out by one-step radiochemical synthesis with a tosylate precursor. The radiochemical yields were low (5–20%) and the synthesis time was 60 min. In this method also, considerable efforts were required to ensure the purification using high performance liquid chromatography (HPLC) causing long synthesis times and low yields. In 2008, the first automated synthesis of [^18^F]fallypride using only SPE purification was reported by Yang et al. with low radiochemical yield of only 15% (Yang et al. 2008). The same authors filed a patent for an almost similar method (Yang et al. 2008) Unfortunately, the patent contains limited information on the synthesis, purification method and quality control and is therefore insufficient to assess whether this method essentially complies with current Good Manufacturing Practice guidelines (GMP). Another patent from 2011 describes the syntheses of [^18^F]fallypride with low base concentration which is advantageous to enhance the radiochemical yield up to 68% and shorten the synthesis time to 51 min by using HPLC for purification (Kim et al. 2011). Some syntheses were carried out using microreactors, which offered the advantage of smaller volumes, especially regarding to HPLC purification of the crude product. For example, Lu et al. were able to produce up to 55.5 MBq of [^18^F]fallypride in 88% yield using a single microreactor (Lu et al. 2009). Lahdenpohja et al., on the other hand, have obtained 3.2 GBq fallypride with 11% radiochemical yield using the microfluidics based iMiDEV™ labelling platform. Other researchers were able to produce up to 7.2 GBq of [^18^F]fallypride in 57% yield using a double microreactor (Wang et al. 2020). The highest molar activity of 730 GBq/µmol [^18^F]fallypride with maximum activity of 555 MBq (65%) could be obtained using microfluidic synthesis (Javed et al. 2014).

The highest yield (68% decay corrected) with activities above 10 GBq was reported by Moon, et al. (Seok Moon et al. 2010). They carried out the synthesis by heating of 2 mg of tosyl-fallypride and [^18^F]Fluoride in acetonitrile (1 mL) at 100 ^0^C for 10 min using 10 µL of 40% tetrabutylammoniumbicarbonate (TBAHCO_3_) as a phase transfer catalyst with a total synthesis time of 51 min, including HPLC purification and solid-phase purification for the final formulation. The radiochemical purity was 97%. In all described cases, considerable efforts were required to ensure the purification using HPLC causing long synthesis times and low yields.

Currently, the most synthesis module suppliers are moving from synthesizer with solid tubing to cassette systems. Single-use cassette-based systems allow the radiochemist to purchase pre-configured cassettes and software programs to avoid the intensive cleaning procedures, resulting in simplified operations and faster GMP compliance by eliminating the need for a validated cleaning method (Boschi et al. 2013; Krasikova 2007). Another advantage of the cassette-based systems is that multiple syntheses can be easily performed even for various tracers and with different radionuclides by changing the cassette (Bruton and Scott 2020). At the same time, the use of cassettes provides improved microbiological safety and eliminates the risk of cross-contamination, thereby achieving better GMP compliance (Lepareur 2022; Velikyan 2015).

A constraint of the synthesis of [^18^F]fallypride, even by using cassette-based platforms, is the continued need for HPLC equipment for the purification step. This causes radio syntheses to become more complex and increases the number of required steps. Therefore, the introduction of SPE cartridges for purification instead of HPLC is considered to be a way to improve Radiosynthesis and represents a desirable option to increase future commercialization of radiopharmaceutical cassettes. The aim of our study is to develop a GMP production of [^18^F]fallypride without HPLC purification step by using common commercial synthesizers.

## Materials & methods

Two commercially available synthesizers were used in this study. The first one is the Synthra RNplus research module with fixed tubing operated by the SynthraView software (Synthra, Hamburg, Germany) and the second is AllinOne synthesis module from Trasis with customized single use cassette operated by the software Trasis Supervision^®^ (Trasis, Ans, Belgium). All single-use cassettes and reagent kits for the Radiosynthesis were sterile and manufactured under GMP conditions.

The precursor (S)-N-[(1-allyl-2-pyrrolidinyl)methyl]-5-(3-toluenesulfonyloxypropyl)-2,3-dimethoxybenzamide (tosyl-fallypride) and (S)-N-[(1-allyl-2-pyrrolidinyl)methyl]-5-(3-fluoropropyl)-2,3-dimethoxybenzamide (fallypride) were purchased from ABX (Radeberg; Germany). Sep Pak Light Alumina N Cartridges, Sep Pak Plus C18 Cartridges and Sep Pak Light C18 Cartridges were purchased from Waters (Eschborn, Germany). Other chemicals were purchased from commercial sources and were used without further purification. [^18^F]Fluoride was produced via the ^18^O(p, n)^18^F reaction with a CTI RDS 112 cyclotron (Berlin).

### Automated synthesis of [^18^F]fallypride using allinone modul of trasis

**Fig. 1:**
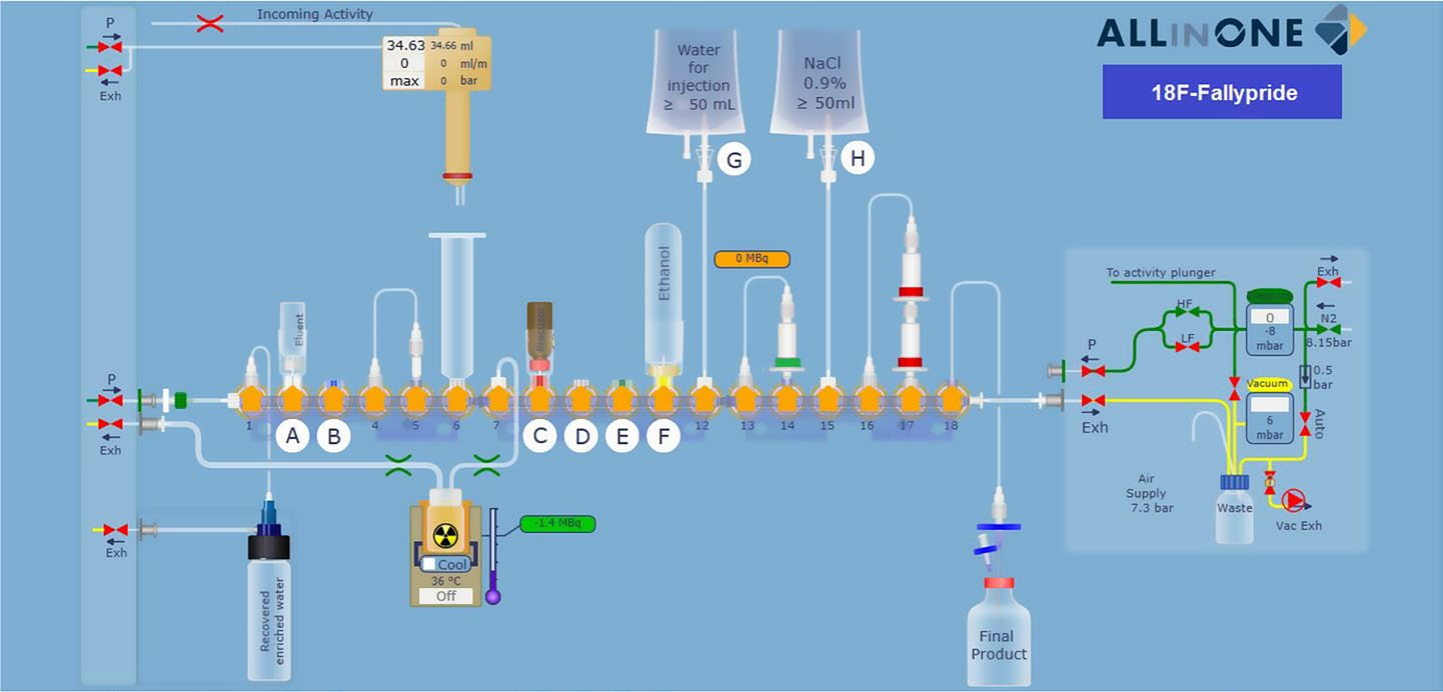
Scheme of the automated synthesizer of [^18^F]fallypride using AllinOne synthesis module

For the fully automated synthesis of [^18^F]fallypride employing the Trasis AllinOne synthesizer, single-use cassettes for the synthesis of [^18^F]-Choline supplied by Trasis (Trasis, Ans, Belgium) were used after modification. As usual with the AllinOne, the cassette was placed on the platform, the reagents loaded on it and the cartridges installed in the correct position as shown in the Fig. 1.

### Automated synthesis of [^18^F]fallypride using RNplus research module of Synthra

**Fig. 2:**
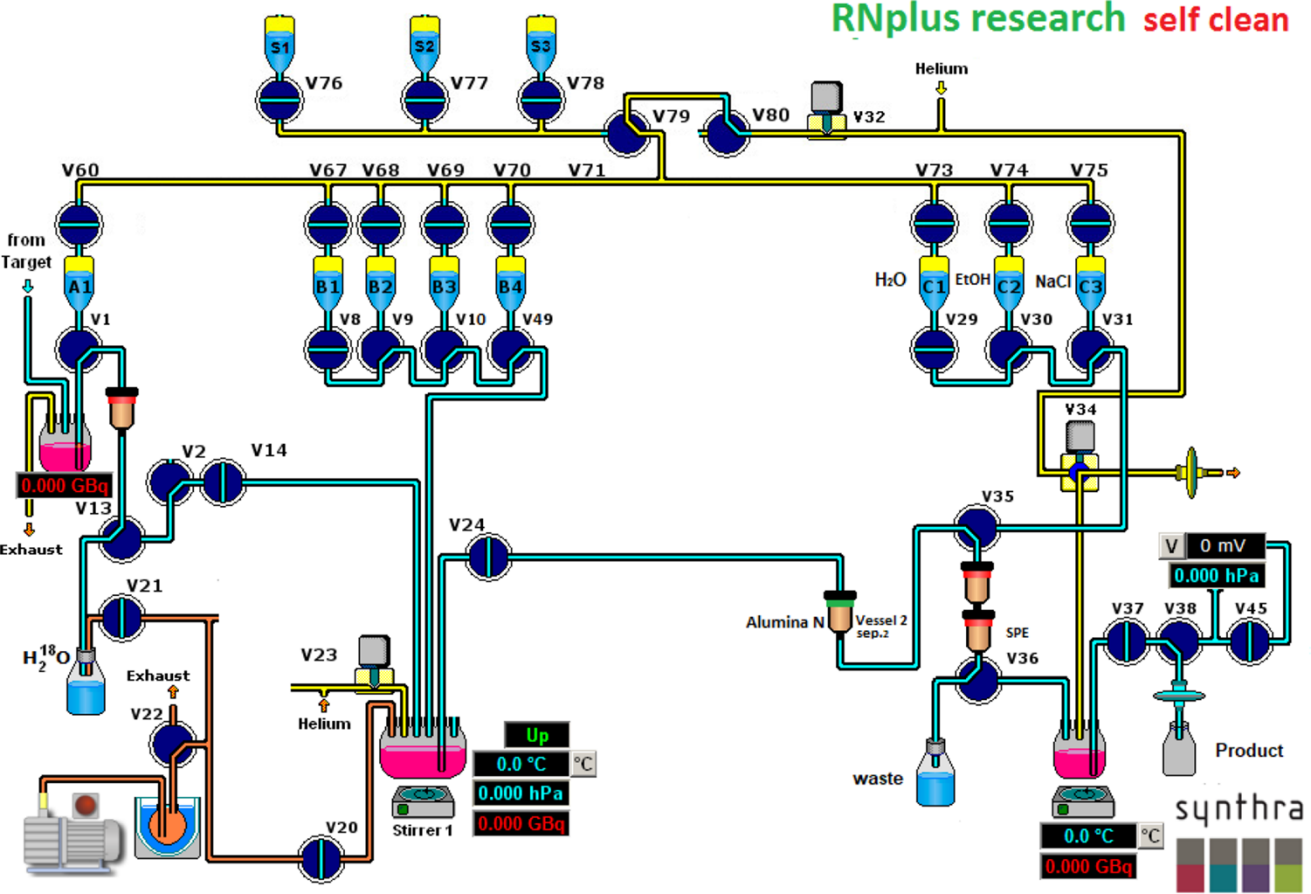
Scheme of the automated synthesizer of [^18^F]fallypride using Synthra RNplus Research module

The RNplus Research module from Synthra shown in Fig. 2 was used for the automated synthesis of [^18^F]fallypride. Prior to the synthesis, the module was cleaned by two automated methods. Firstly, washing with water to remove water-soluble contaminants from the peptide vial and the reaction vessel. Secondly, washing with an organic solvent (ethanol) to rinse all valves, the peptide vial and the reaction vessel. Finally, all module components were dried using nitrogen gas flow.

### Synthesis of [^18^F]fallypride using the phase transfer catalyst system K_2_CO_3_ / Kryptofix-[2.2.2

20–25 GBq (*n* = 3, mean: 20.7 ± 4) [^18^F]fluoride were delivered to the synthesis module (AllinOne or RNplus) as a solution in [^18^O]H_2_O (1.8 mL). This solution was passed through a small anion exchange column (30 mg, HCO_3_-form) to trap the [^18^F]fluoride. The [^18^F]fluoride was then eluted into the reactor using a mixture of a solution of Kryptofix-[2.2.2] (15 mg in 0,8 mL acetonitrile) and a solution of aqueous potassium carbonate (200 µL, 0.1 M). The solvent was then evaporated for 3 min at 70 °C under vacuum with helium stream and further 3 min at 115 °C followed by additional 3 min without helium flow under vacuum. The tosyl-fallypride precursor (2 mg) in acetonitrile (1mL) was then added to the dried [^18^F]KF–K222 and the mixture was heated at 90 °C for 20 min. Afterwards the reactor was cooled to 40 °C. The reaction mixture was then passed through three cartridges connected in series: Alumina N Plus Light, Sep Pak Plus C18 and Sep Pak Light C18 cartridges. The cartridges were then washed with 40 mL of water. [^18^F]fallypride was eluted from the cartridges by 1 mL of ethanol, then passed through a 0.22 μm sterile filter and collected in the product vial before being further diluted with 12 mL of 0.9% aq. NaCl to obtain 6.8 ± 0.9Gbq (*n* = 3) of [^18^F]fallypride ready for human use in approximately 40 min (yield = 31 ± 3%, not decay corrected). The radiochemical purity was 98.9 ± 0.34%.

### Synthesis of [^18^F]fallypride using the phase transfer catalyst system TBAHCO_3_

2–5 GBq (*n* = 3, mean: 3.2 ± 1.4) [^18^F]fluoride were delivered to the synthesis module (AllinOne or RNplus) as a solution in [^18^O]H_2_O (1.8 mL). This solution was passed through a small anion exchange column (30 mg, HCO_3_^-^form) to trap the [^18^F]fluoride. Then it was eluted to the reactor with 10µL of 40% TBAHCO_3_ in EtOH/H_2_O (1.0/0.2 mL). The eluate was evaporated to dryness in the reactor at 95 °C within 6 min under vacuum and a stream of helium.

Tosyl-fallypride (2 mg) in acetonitrile (1mL) were then added to the reactor and the mixture was heated at 95 °C for 10 min. Afterwards the reactor was cooled to 40 °C. The reaction mixture was then passed through three cartridges connected in series: Alumina N Plus Light, Sep Pak Plus C18 and Sep Pak Light C18 Cartridges. The cartridges were then washed with 40 mL of water. The [^18^F]fallypride was eluted from the cartridges by 1 mL of Ethanol, passed through a 0.22 μm sterile filter and collected in the product vial before being further diluted with 12 mL of 0.9% aq. NaCl to obtain 1.9 ± 0.Gbq (*n* = 3) of [^18^F]fallypride ready for human use in approximately 28 min (yield = 59 ± 4%, not decay corrected). The radiochemical purity was 98.4 ± 0.29%.

### [^18^F]fallypride quality control

The quality control (QC) procedures performed for [^18^F]fallypride are based on the current requirements for radiopharmaceuticals set out in the European Pharmacopoeia for the manufacture of radiopharmaceuticals (Radiopharmaceutical Preparations-04/2023:0125) and the quality control release criteria and are summarized in Table 1.

**Table 1:** Summary of the product specifications and validation results for three consecutive productions for [^18^F]fallypride. RRT: relative retention time.*: detection limit GC = 10 Ppm; **: detection limit endotoxin = 5 EU/mL; ***: detection limit spot test ≤ 50 µg/ml for Kryptofix and ≤ 260 µg/ml for TBA+

QC Test	Release Criteria	[^18^F]Fallypride with TBA^+^ (*n* = 3)	[^18^F]fallypride with Kryptofix (*n* = 3)
Yield %	N/A	59%	31%
Visual Inspection	Clear, colorless	Clear, colorless	Clear, colorless
Radiochemical Identity	RRT = 0.9–1.1	1.01	1.00
Radiochemical Purity	> 95%	98.4	98.9
Chemical purity	Precursor	≤ 1 µg/ml	≤ 1 µg/ml
	non-radioactive impurities	396 µg	396 µg
Residual Solvent* Analysis	Aceton < 5000 ppm	Not detected	Not detected
	Ethanol No limit established	7,7%	7,7%
	Acetonitrile ≤ 410 ppm	1.8	1.8
Dose pH	4.5–7.5	6.8	6.5
Residual (spot test)**	Kryptofix ≤50 µg / mL TBA^+^≤260 µg/mL	Passed**	Passed**
Sterile Filter Integrity Test	> 3,2 bar	3.6	3.7
Radionuclidic Identity (t_1/2_)	105–115 min	109 min	108 min
Endotoxin Analysis***	≤17.5 EU/mL	Passed	Passed
Sterility Testing	No colony growth out to 14 days	passed	passed

### Radionuclidic identity and purity of ¹⁸F

The radionuclidic identity and purity of ¹⁸F were determined using gamma spectroscopy with High Purity Germanium (HPGe) radiation detector (Ortec, Oak Ridge, TN, USA). To confirm the radionuclide identity, a 10 µL sample of a 1:50 dilution of the product (5–30 kBq) were analyzed using the gamma spectrometer. The detected gamma photons must have an energy of 0.511 MeV, corresponding to the positron annihilation of ¹⁸F.

The half-life of ¹⁸F was determined by measuring the decay of the same sample under identical geometric conditions at five time points, with 20-minute intervals between measurements. The calculated half-life must be within the expected range of 105–112 min.

For radionuclidic purity assessment, the same sample was remeasured 48 h after initial analysis using the gamma spectrometer under identical geometric conditions. This measurement was performed to detect any radionuclidic impurities with a half-life longer than 5 h. The remaining activity at 48 h must be < 0.001% of the initial activity to ensure high radionuclidic purity.

### Radiochemical identity

Radiochemical identity was determined using HPLC analysis System (Gold HPLC System, Beckman Coulter, USA) fitted with an UV detector and HERM LB 500 γ-detector (Berthold Technologies GmbH & Co. KG, Germany). The HPLC column was Chromolith HR RP-18 100 × 3 mm (Merck KGaA, Germany), Solvents: A: H_2_O (0.1% TFA); B: acetonitrile (0.1% TFA); gradient: 0–8 min 0-100%B; flow rate: 2.0 mL/min).

**Fig. 3:**
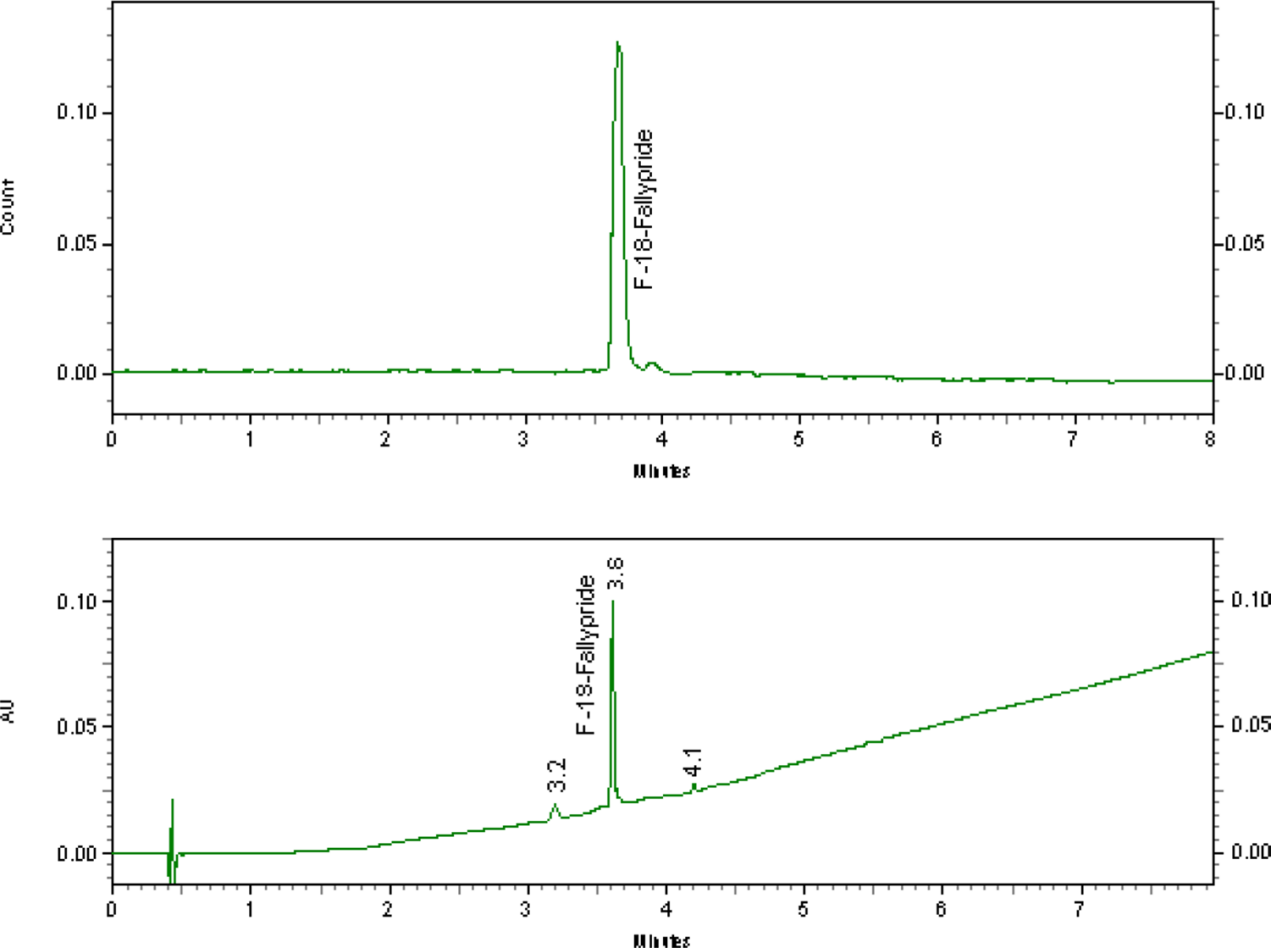
Chemical and radiochemical purity detection of [^18^F]fallypride analized via HPLC with UV detector at 254 nm and radioactivity detector (NaI) showing [^18^F]fallypride and the added [^19^F]fallypride standard. HPLC conditions: Chromolith HR RP-18 100 × 3 mm, Solvents: A: H2O (0.1% TFA); B: acetonitrile (0.1% TFA); gradient: 0–8 min 0-100%B; flow rate: 2.0 mL/min)

To identify [^18^F]fallypride the retention time of the radioactive Peak (Rt) was compared with the Rt of the reference standard [^19^F]fallypride (purchased from ABX GmbH, Germany). A relative retention time (RRT) of 1 ± 0.1 indicates that the radioactive compound was identical to the reference compound, confirming its radiochemical identity. Representative analytical HPLC traces are displayed in Fig. 3.

### Chemical purity

Visual inspection of the final [^18^F]fallypride solution was conducted to ensure its clarity, color, and the absence of particles. The solution was examined under proper lighting conditions against white light and black background. It must appear as a clear, colorless liquid with no visible particles or turbidity.

Chemical purity was determined by HPLC and liquid chromatography–mass spectrometry (LC-MS) consisting of Agilent 1100 HPLC with UV and radioactivity detector (GinaStar Elysia Raytest, Germany) and Quattro Premier XE mass spectrometer (Waters, USA). The HPLC column was Chromolith HR RP-18 100 × 2 mm (Merck KGaA, Germany), Solvents: A: H_2_O (0.1% TFA); B: acetonitrile (0.1% TFA); gradient: 0–8 min 0-100%B; flow rate: 0.5 mL/min).

### Residual solvent analysis

The residual solvent levels like acetone from the drying process, ethanol and acetonitrile in [^18^F]fallypride doses were analyzed using a Varian 3500 GC gas chromatograph. The analysis was performed with an Rtx-200 column (0.25 mm ID, 30 m) using nitrogen as the carrier gas at a flow rate of 0.95 mL/min. The injector temperature was set to 250 °C, and the temperature program was 50 °C (held for 5 min), followed by an increase of 10 °C/min.

### TLC analysis

The thin layer chromatography analysis (TLC) of the radiochemical purity was conducted using an Elysia-Raytest linear analyser detector RITA (Elysia-Raytest GmbH, Germany). Radio TLC was performed on Tec-Control Dark Green (Biodex Medical Systems, USA) developed with acetonitrile − 0.9% NaCl (1v/1v).

### Sterile filter integrity test

For testing the filter for integrity, the used and still wet filter is connected to a cannula at the outlet and to a nitrogen line at the filter inlet. The cannula tip must be submerged in water in a container during the test. The nitrogen supply is then switched on and the pressure is slowly increased via the pressure control valve until rapid, continuous bubble formation is observed at the cannula outlet. If the bubble point determined in this way is above 3.2 bar, the filter is considered functional (Table 1).

### Dose pH

To determine the pH value of the product, commercially available pH test strips (pH-Fix 2.0–9.0, color-fixed, non-bleeding from Macherey-Nagel, Germanny) are used. 20 µl of the product solution is applied to a test strip. The colors are compared with the color chart, and the pH value that matches closest is read off. As listed in Table 1, the pH was ranging between 6.5 and 7.5.

### Endotoxin analysis

The endotoxin content in the synthesized [^18^F]fallypride was analyzed using a Endosafe^®^ nexgen-PTS™ from Charles River. Doses had to contain ≤ 175 Endotoxin Units (EU) / mL to be deemed acceptable. Limulus-amoebocyte-lysate (LAL) test for bacterial endotoxin resulted < 17.5 EU/mL in all analyzed samples (Table 1).

### Sterility testing

To verify sterility 25 µL probes of the product solution were added to Liquid thioglycolate medium plates (FTM) and to plates containing soybean casein digest agar medium (SCDM) to determine possible anaerobic, aerobic, and microaerophilic as well as fastidious and non-fastidious microorganisms contamination respectively. The plates were then incubated for 14 days together with positive and negative controls at 32 °C for FTM and 22 °C for SCDM plates according to the European Pharmacopoeia (Ph. Eur.). After specified intervals (day 3, day 8, and day 14), the plates are visually inspected. Positive samples must show culture growth (detectable by turbidity), while negative samples must show no growth at all within the entire 14-day test period to be considered sterile.

All samples met the sterility specifications (Table 1).

### Thin layer chromatography spot test for determination of kryptofix-[2.2.2] and TBAHCO_3_ content

The Kryptofix-[2.2.2] content was determined as described by (Tanzey et al. 2020) by using the thin layer chromatography technique on POLYGRAM^®^ SIL G / UV254, 4 × 8 cm plate using a mixture of methanol and 25% ammonium hydroxide solution in water (90:10 v / v) as the mobile phase.

Spots of 1,0 µL of standard solution of Kryptofix-[2.2.2] or TBAHCO_3_ in the concentration of 50 µg/mL, sample of [^18^F]fallypride, solvent (water: EtOH: 9:1) and positive control (500 µg/mL) were applied established 1 cm from the lower edge of the chromatographic sheet by means of an Eppendorf micro pipette. The samples were then dried with the aid of a hot air blower. The sheet was developed with the mobile phase and the dried sheets were then placed on a holder inside a glass chamber homogenously saturated with iodine vapor (ca. 5 gr) for 1 min.

Any spot had not to be more intense than the reference solution, consequently containing only an equal or lower than 50 µg/mL of Kryptofix-[2.2.2] or TBAHCO_3_ to meet Ph. Eur. requirements for the limit of Kryptofix-[2.2.2] impurities in ^18^F-labelled radiopharmaceuticals.

## Results

**Fig. 4:**
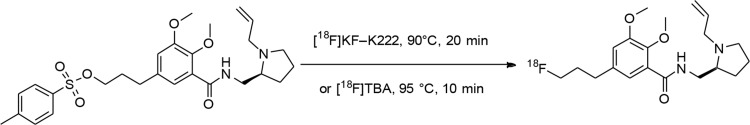
Scheme of the synthesis of [^18^F]fallypride

**Fig. 5:**
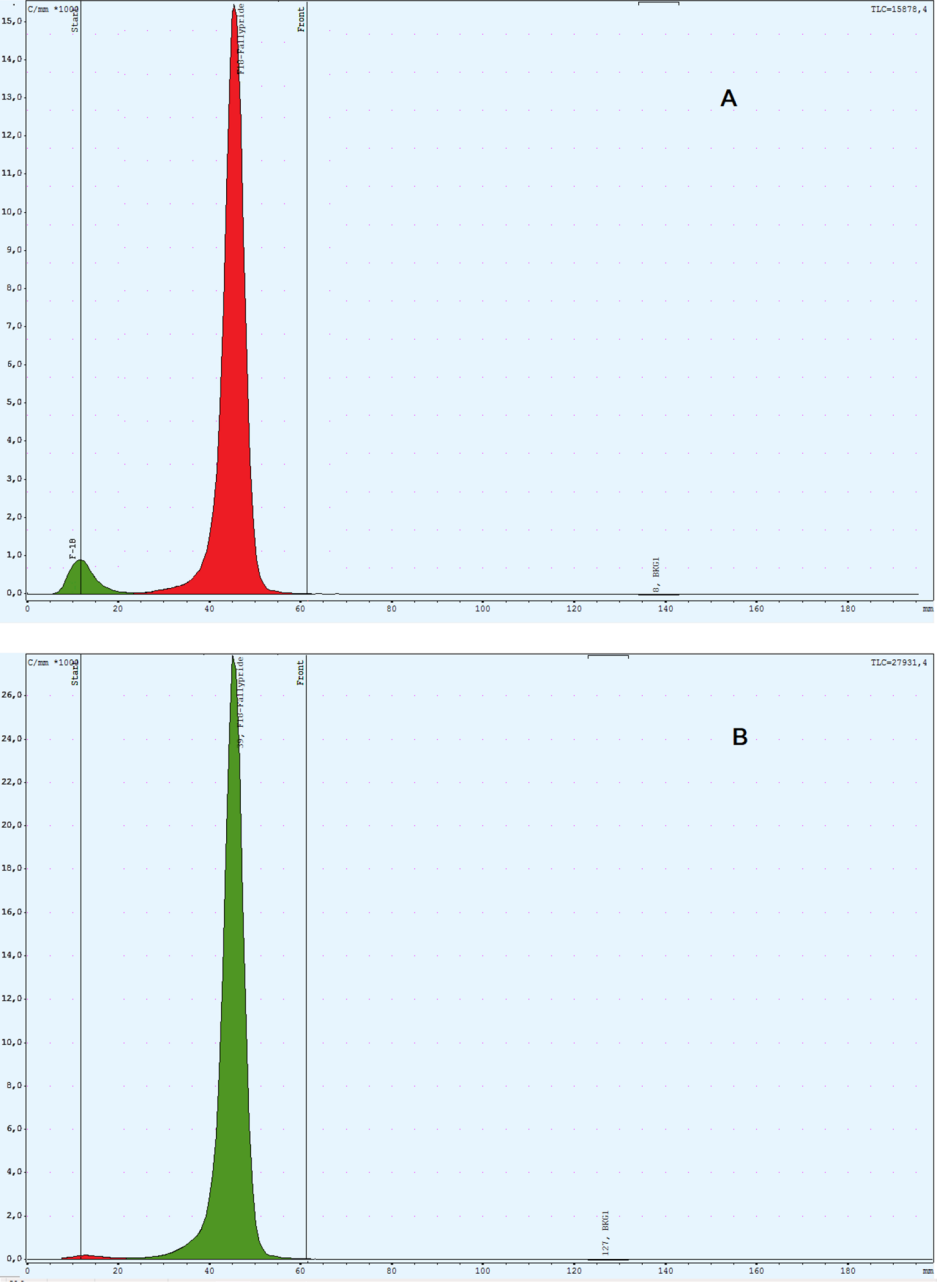
Radiochemical purity analyzed with radio TLC scanner. A: for [^18^F]fallypride purified using only a Sep-Pak plus C-18 cartridge. B: the same as A, but with addition of a Sep-Pak Alumina-N cartridge and Sep Pak Light C18 Cartridge

An improved GMP compliant synthesis method for [^18^F]fallypride has been developed for both the cassette based synthesis system AllinOne (AiO) and the research synthesis module with fixed tubing (RNplus). [^18^F]fallypride could be obtained ready for human use as physiological solution after purification on Alumina N Plus Light, Sep Pak Plus C18 and Sep Pak Light C18 cartridges and formulation with 1mL ethanol Fig. 4. The Radiochemical purity was 98.9 ± 0.34% (*n* = 3) and 98.4 ± 0.29% (*n* = 3) for the synthesis with kryptofix-[2.2.2] and TBAHCO_3_ respectively. and no contamination of the sterile solution with chemicals or solvents used during the synthesis was detected (Table 1). Representative chromatograms of radio HPLC and radio TLC are presented in Figs. 3 and 5b. Repeated analyzes up to 4 h after synthesis showed that the radiochemical purity was still > 95%.The method involves the abandonment of HPLC purification in favor of a faster and more convenient SPE purification method that led to [^18^F]fallypride as physiological solution ready for human use meeting all required specifications for radiopharmaceuticals.

For both synthesizers (AiO and RNplus) the optimization of the synthesis parameters in combination with the SPE cartridge purification results in [^18^F]fallypride in high radiochemical yield (59% not decay corrected) in only 28 min when using TBAHCO_3_ as phase transfer catalyst system and 31% (not decay corrected) in 40 min when using K_2_CO_3_ / Kryptofix-[2.2.2] (Table 1).

**Fig. 6:**
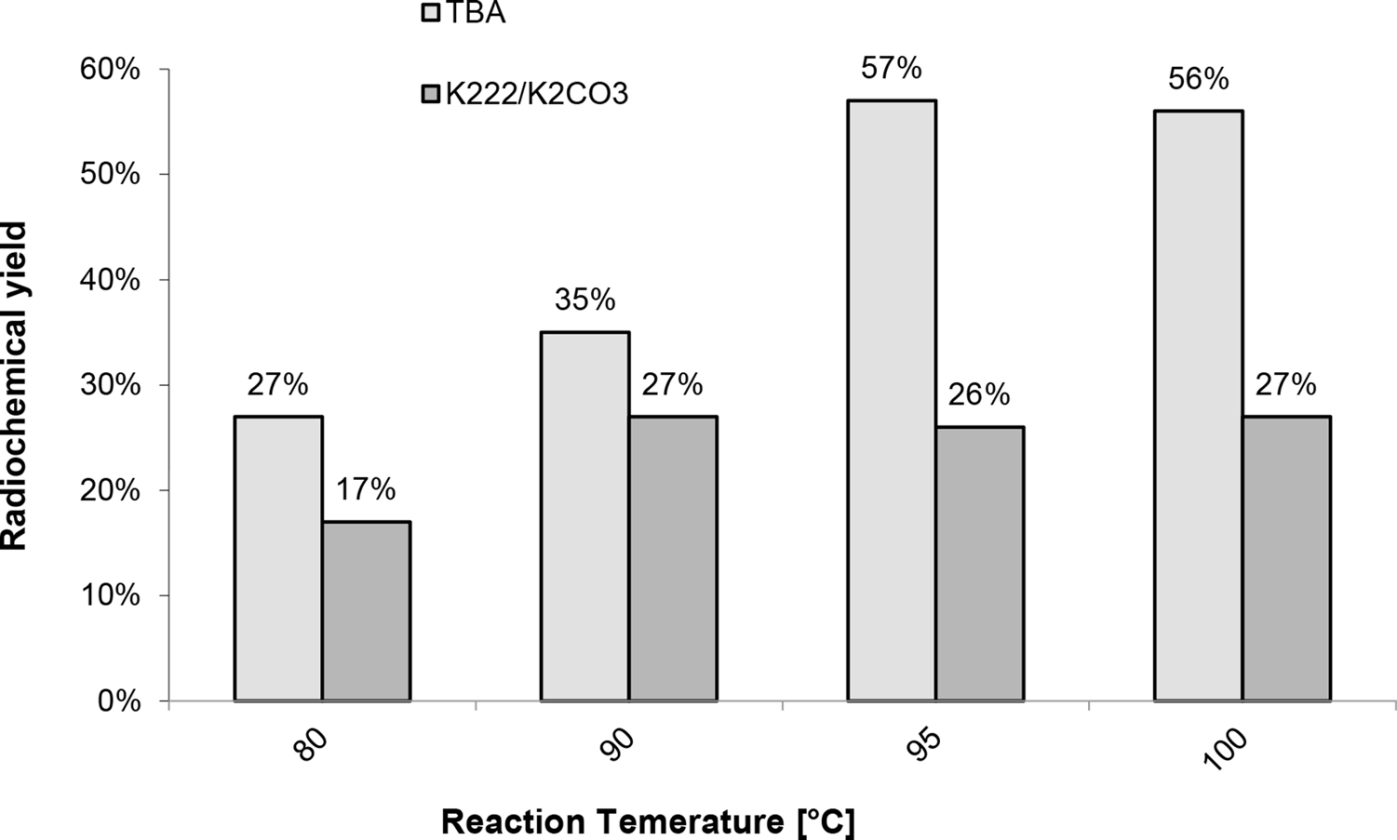
Radiochemical yield of [^18^F]fallypride at versus temperature using 2 mg precursor and 10 min reaction time with TBAHCO_3_ as well as 20 min reaction time with K_2_CO_3_/Kryptofix-[2.2.2]

**Fig. 7:**
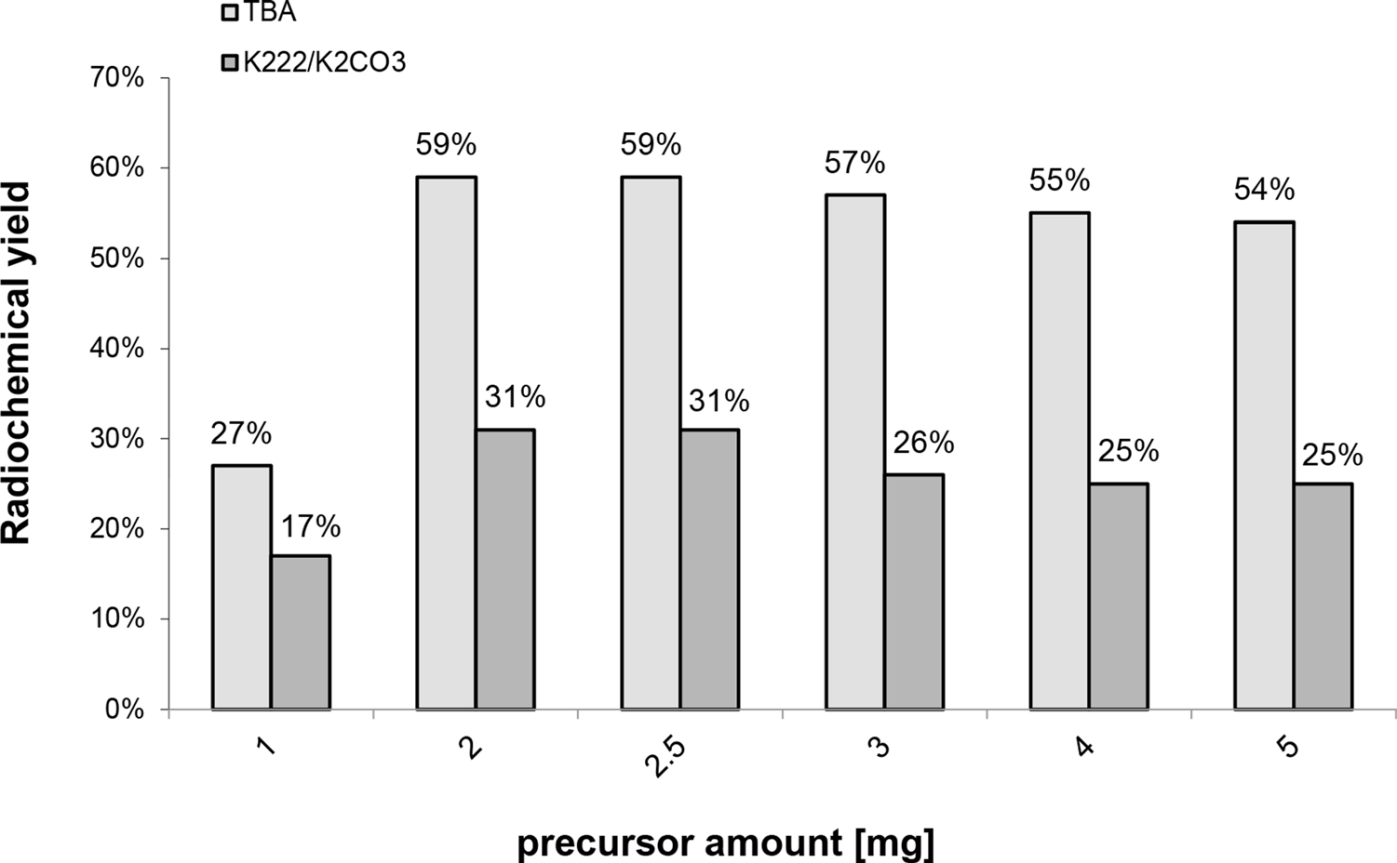
Dependence of the radiochemical yield of [^18^F]fallypride on the quantity of precursor for 10 min reaction time with TBAHCO_3_ and 20 min with K_2_CO_3_/Kryptofix-[2.2.2] at reaction time 95 °C

**Fig. 8:**
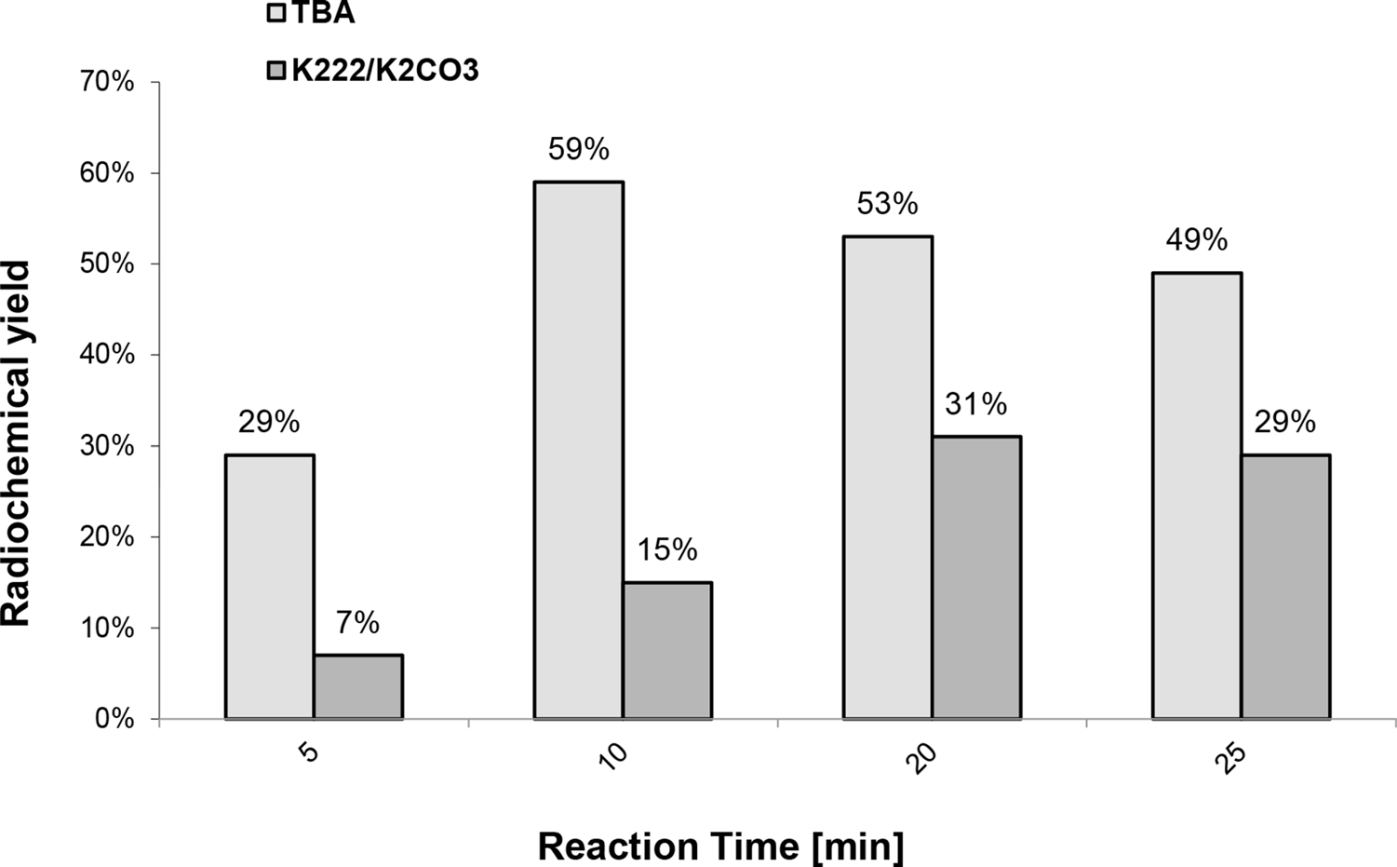
Dependence of the radiochemical yield of [^18^F]fallypride on reaction time using 2 mg of precursor at 95 °C

The studies on the effect of the reaction temperature (Fig. 6), precursor amount (Fig. 7) and reaction time (Fig. 8) show that the maximum radiochemical yield is reached at 95°, with 2 mg precursor and 10 min reaction time.

**Fig. 9:**
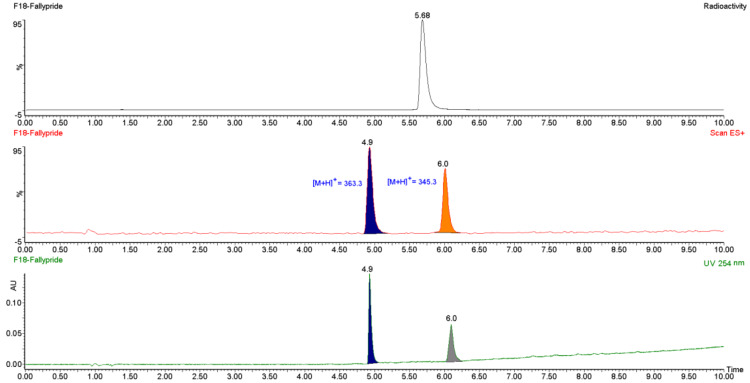
LC-MS analysis of the final solution of [^18^F]fallypride with simultaneous recording of mass, radioactivity and UV absorption at 254. HPLC: Chromolith HR RP-18 100 × 2 mm (Merck KGaA, Germany), Solvents: A: H^2^O (0.1% TFA); B: acetonitrile (0.1% TFA); gradient: 0–8 min 0-100%B; flow rate: 0.5 mL/min)

## Discussion

In recent decades there has been a shift in the field of automated synthesis modules for radiopharmaceuticals from the fixed tubing system, in which the liquid flow is regulated by inert gas, to a sterile disposable cassette system, in which the liquid flow is regulated by a syringe pump. One of the key advantages of the cassette system approach is that it provides greater microbiological safety and eliminates the risk of cross-contamination compared to the fixed tube approach. In this way, the GMP requirements are met better (Bruton and Scott 2020). However, cassette systems also have some disadvantages compared to fixed tubing systems. The main disadvantage of using disposable cassettes is their high cost and the dependency on the cassette manufacturers. Though, for both systems, in many cases, purification of the product using HPLC is necessary, which means additional complications and long synthesis times. In these cases, the possibility of using SPE cartridges instead of HPLC is highly desirable as it significantly simplifies the synthesis and increases flexibility.

In a previous work by Moon et al. from 2010 the Radiosynthesis of [^18^F]fallypride was carried out by a labeling reaction using the phase transfer catalyst TBAHCO_3_ (Seok Moon et al. 2010). They reported maximum radiochemical yield of approximately 50% (68% decay corrected) at 100 °C and a reaction duration of 51 min. Due to the need to use HPLC for the purification of the final product, this method is quite complex and needs long syntheses time. This circumstance may limit the application of [^18^F]fallypride in clinical practice and leads to relatively high dose cost rate. For this reason, we turned our attention to develop a simplified fast and high yielding automated synthesis using appropriate cartridges for the purification instead of an HPLC run.

Using the Synthra RNplus research module with fixed tubing, we started with the synthesis of [^18^F]fallypride described by Moon et al. (Seok Moon et al. 2010) and replaced the HPLC purification by a Sep-Pak plus C-18 cartridge purification as described by Yang et al. (2008). However We found out that the recovery of [^18^F]fallypride was only 85%. Accordingly, 15% of the product was not retained by the cartridge and was lost as it was eluted through the Sep-Pak plus C-18 cartridge into the waste. In addition, the determined radiochemical purity using radio TLC analysis reveals a radiochemical purity of only 94% [^18^F]fallypride (Fig. 5a). For this reason, we introduced two modifications to the purification process. Firstly, we added a Sep-Pak Alumina-N cartridge as this was suggested to eliminate the non-reacted [^18^F]fluoride (Shao et al. 2011) and secondly, we added a second Sep Pak Light C18 cartridge to increase the trapping efficiency of [^18^F]fallypride and minimize its loss of into the waste. In this way the loss of [^18^F]fallypride into the waste was < 2% and the amount of [^18^F]Fluoride in the final product was reduced to 1.3 ± 0.41% (*n* = 6) (Figs. 3 and 5b).

Next, we focused on optimizing the labelling conditions to increase the radiochemical yield and to reduce the reaction time by optimizing the reaction temperature. We carried out two series of syntheses under different reaction temperatures using both the phase transfer catalysts systems TBAHCO_3_ and K_2_CO_3_/Kryptofix-[2.2.2]. The results are shown in Fig. 6 and show an optimal reaction temperature of about 95 °C.

The influence of precursor amounts on yields was then subsequently studied with five different precursor amounts of 1 mg, 2 mg, 2,5 mg, 3 mg, 4 mg and 5 mg at a reaction temperature of 95 °C. The results are represented in Fig. 7. The maximum yield of [^18^F]fallypride was achieved when using 2 mg of precursor. The radiochemical purity was 98.9%. If 2.5 mg or more of precursor were used, a small peak at 3.9 after the peak of [^18^F]fallypride (Rt = 3.7) was recorded in the radioactive chromatogram, as shown in Fig. 3. This peak increased to about 3% of the total radioactivity when more than 3 mg of precursor was used. The formation of radioactive byproduct detected at higher retention time as [^18^F]fallypride is described by Wang et al. and Huhtala et al. as a result of radiolysis due to high activity concentration during the synthesis (Huhtala et al. 2019; Yang et al. 2008). The use of higher precursor amounts by Gao et al. led to a reduction in radiochemical yield and chemical purity (Gao et al. 2010). For this reason, the amount of precursor should not exceed 2 mg.

Applying the already found best conditions for the reaction temperature and precursor amounts (95 °C with TBAHCO_3_; 90 °C with K_2_CO_3_/Kryptofix-[2.2.2]; 2 mg precursor) the effect of the reaction time was then examined. The experiments showed an optimal reaction time of 10 min with TBAHCO_3_ and 20 min with K_2_CO_3_/Kryptofix-[2.2.2] (Fig. 8).

As can be seen in Fig. 3, the UV trace at 254 nm shows two non-radioactive peaks besides the peak of the added [^19^F]fallypride standard peak. To identify the corresponding compounds, we performed additional analysis using LC-MS with simultaneous recording of mass, radioactivity and UV absorption at 254. The results in Fig. 9 show two peaks in the MS trace corresponding to the masses [M + H]^+^=363.22 (Rt = 4.9) and [M + H]^+^=345.20 (Rt = 5.9). The first is the hydrolysis product of the tosyl-fallypride to the corresponding alcohol and the second is the elimination product, respectively (Table 2). The same byproducts were observed by Mukhejee et al. (Mukherjee et al. 1995).

**Table 2:**
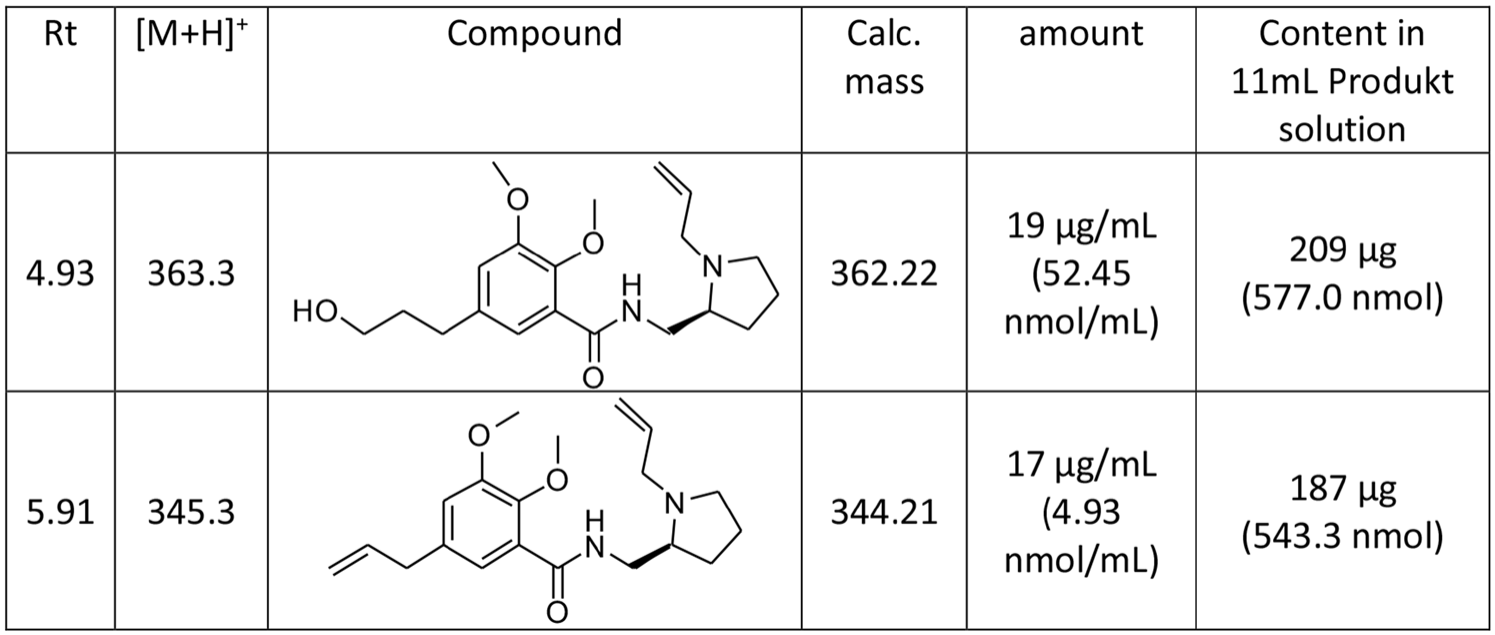
Detected non-radioactive compounds using LC-MS analysis in the [^18^F]fallypride final solution

Since no signal was detected by LC-MS for tosyl-fallypride and [^19^F]fallypride we concluded that the concentration of both compounds in the final product solution is below the MS detection limit. The detection limit determined previously by calibrating the MS with several concentrations (0.01–200 µg/mL) was found to be 0.1 µg/mL. Based on this concentration and the activity at the end of the synthesis we calculated a molar activity 39.1-256.1 GBq/µmol, which is comparable to molar activities in der literature 37.3–62.9 GBq/µmol (Mukherjee et al. 1995), 81 − 27 GBq/µmol (Wang et al. 2020), 15–78 GBq/µmol (Lazari et al. 2014) and 140–192 GBq/µmol (Seok Moon et al. 2010).

Since GMP regulations for radiopharmaceuticals tend to support single-use cassette systems, cartridge cleaning instead of HPLC cleaning in these systems simplifies operations and facilitates GMP compliance. In addition, the use of synthesizers with disposable cassettes increases reliability and enables rapid training of production personnel. Therefore, we transferred the conditions for the [^18^F]fallypride synthesis obtained with the Synthra RNplus Research module to the AllinOne module from Trasis. The [^18^F]fallypride synthesis with the AllinOne module applying these conditions and using the parameters as shown in Fig. 1 gave the same results as with the RNplus Research module.

## Conclusion

We could successfully demonstrate that the synthesis of [^18^F]fallypride is practicable on commercial cassette-based systems as well as on customized modules with fixed tubing systems without the need for HPLC purification. The fully automated protocols are in accordance to GMP standards and allow the synthesis of [^18^F]fallypride without HPLC within 28 min including purification and formulation steps with high labeling yield as well as high Radiochemical and radionuclide purity.

## Data Availability

All data generated or analyzed during this study are included in this published article.

## Abbreviations

GMP: Good manufacturing practice
HPLC: High performance liquid chromatography
PET: Positron emission tomography
Ph Eur: European pharmacopeia
QC: Quality control
TLC: Thin-layer chromatography
